# “I Will Meet the Patient Wherever They Are At:” Using Structural Competency and Simulation to Improve Attitudes and Practices Towards Structurally Vulnerable Patients

**DOI:** 10.1007/s40670-026-02932-z

**Published:** 2026-10-23

**Authors:** Sydney Katz, Mary Ann Hernando, Erika Abramson, Orysya Soroka, Deborah Ofosu, Amanda Ramsdell

**Affiliations:** 1 Division of General Internal Medicine, Weill Cornell Medical College, New York, NY USA; 2 Department of Pediatrics, Weill Cornell Medical College, New York, NY USA; 3 Kent State University College of Podiatric Medicine, Independence, OH USA; 4 Department of Medicine, Division of General Internal Medicine, Section of Hospital Medicine, Weill Cornell Medicine | NewYork-Presbyterian Hospital, 525 East 68th Street, Box 331, New York, NY 10065 USA

**Keywords:** Structural determinants of health, Health equity, Patient discharge, Medical education

## Abstract

**Introduction:**

Structurally vulnerable patients experience worse health outcomes, exacerbated by healthcare workers’ negative attitudes and limited understanding of structural barriers to care. Structural competency may improve attitudes towards structurally vulnerable patients, but the impact on attitudes and practices has not been rigorously evaluated, particularly in clinical settings.

**Methods:**

Medical students at Weill Cornell Medical College in New York City participated in a structural competency-informed curriculum aligned with transformative learning theory which incorporates clinical skills training with simulation and reflection during academic years 2022–2023 and 2023–2024. Authors conducted a convergent mixed-methods study using pre-post surveys and thematic analysis of reflections to explore if and how the curriculum impacted students’ attitudes and practices towards structurally vulnerable patients. Surveys included the Medical Condition Regard Scale (MCRS) and items accessing self-efficacy in applying the curriculum’s learning objectives.

**Results:**

Of 190 students, 77 (40.5%) completed the pre-survey and 50 (26.3%) completed pre- and post-surveys. The mean pre-course MCRS score (*n* = 50) was 4.18/6 (SD 0.79) and the mean post-course MCRS score was 4.57/6 (SD 0.75), showing a mean increase of 0.39 (SD 0.49, t-test *P* < 0.001, Cohen’s *d* = 0.81). Thematic analysis of 21 reflections identified four themes highlighting how students employed patient-centered communication strategies, utilized best practices in discharge planning, engaged in critical self-reflection and reframing, and appreciated how structural barriers shape health.

**Conclusion:**

Findings suggest that integrating education on structural factors with clinical skills training, reinforced through simulation and reflection, can improve self-reported attitudes and perceived self-efficacy in caring for structurally vulnerable patients.

## Introduction

Structural vulnerability, defined as susceptibility to harm due to socioeconomic, political, and cultural forces [1], has been associated with worse health outcomes [2–4]. Studies have suggested that poor outcomes may be exacerbated by healthcare workers’ negative attitudes toward structurally vulnerable patients and limited understanding of structural barriers to care, like lack of insurance and fragmented healthcare access [5, 6]. Despite these negative outcomes, and the potential to improve patient outcomes by increasing clinicians’ regard toward structurally vulnerable patients, evidence-based curricula to prepare trainees to address the needs of this population are limited.

Structural competency (SC) has been proposed as a promising educational framework that can help reframe healthcare workers’ understanding of the structural factors that may impact patients’ health outcomes [7] and has been described as “the ability of providers to conceptualize social and structural barriers to health and incorporate awareness of these barriers into clinical care [8].” Preliminary qualitative findings suggest SC curricula may improve healthcare workers’ attitudes toward structurally vulnerable patients and reframe how they think about this population [9, 10]. However, the impact of SC curricula on clinician attitudes and practices has not been rigorously evaluated, particularly in the authentic or simulated clinical setting.

We previously described the pilot development of an Objective Structured Clinical Examination (OSCE) embedded in a required Health Policy course which was informed by the SC framework [11] and aligned with the principles of transformative learning (TL) theory, an adult learning theory which explains how learners make fundamental shifts in their worldview through critically examining previously held assumptions [12]. During the simulated clinical encounter, a patient requests to leave against medical advice (AMA). AMA discharge is more common among patients experiencing structural vulnerabilities such as lack of health insurance, homelessness, substance use disorders, and mental illness [13] and has been historically attached to stigmatization and conflict with the healthcare system [14]. Given the strong link between structural factors and the risk of leaving the hospital AMA, we selected this clinical scenario to serve as a proxy for learners to navigate structural barriers to care. We found that teaching SC through simulation was feasible and well-accepted by students [11].

In this study, we build upon our prior work by using a mixed-methods approach to investigate whether this novel curriculum, which was informed by the SC framework and incorporates simulation and active reflection, can change self-reported attitudes and intended practices toward structurally vulnerable patients – and, if so, how. We hypothesized that medical students’ self-reported attitudes toward structurally vulnerable patients would improve after participating in this structural competency-informed curriculum aligned with the principles of transformative learning, and that students would report increased comfort applying structurally informed approaches in their clinical practice.

## Materials & Methods

### Study Design

We conducted a convergent mixed-methods study using pre-post surveys and qualitative analysis of student reflections to evaluate a structural competency-informed OSCE curriculum which incorporates simulation and guided reflection. Mixed-methods approaches are well suited for studying complex educational interventions in natural settings [15]. By integrating quantitative and qualitative findings, we aimed to generate more comprehensive insights into the curriculum’s impact on trainee attitudes and practices.

### Curriculum Overview

The OSCE curriculum [11] aims to bring a clinical context to a classroom-based course which uses the SC framework to teach health equity by equipping learners with the skills to care for structurally vulnerable patients. Additionally, it provides a standardized experience for students to apply course didactics and elements of the SC framework to clinical care.

Curricula include didactics on the SC framework, health policy topics, and clinical skills important for serving a structurally vulnerable population such as harm reduction strategies and trauma-informed care. The course culminates in an OSCE where students portray an intern caring for a structurally vulnerable patient who requests to leave the hospital AMA. After the OSCE, students complete written reflections and participate in a small group debrief. Learning objectives for the curriculum include using patient-centered communication skills, employing harm reduction strategies while creating a discharge plan, providing patient-centered discharge education, and identifying structural factors which contribute to a patient leaving prematurely (Table 1).

**Table 1:** OSCE curriculum learning objectives, research question, and methods of assessment used for advanced medical students, Weill Cornell Medical College, New York, New York, 2023–2024

Descriptor	Details
Learning objectives for OSCE	• Student will use patient-centered communication skills during a premature/AMA discharge encounter • Student will use harm reduction strategies while creating a premature/AMA discharge plan • Student will complete a patient-centered discharge education encounter • Student will identify and reflect on structural factors that increase a patient’s likelihood of leaving prematurely/AMA and may impact the care received by a patient who leaves prematurely/AMA
Structural Competency Framework Core Competencies (Metzl 2014)	• Recognizing the structures that shape clinical interactions • Developing an extra-clinical language of structure • Rearticulating “cultural” formulations in structural terms • Observing and imagining structural interventions • Developing structural humility
Research Question	Can a novel curriculum, which was informed by the SC framework and incorporates simulation and active reflection, change attitudes and intended practices toward structurally vulnerable patients – and, if so, how?
Methods of Assessment	
Pre-/Post-Course Quantitative Surveys	− 11-point Medical Condition Regard Scale towards patients who leave against medical advice *(attitudes)* − 4 Likert scale self-efficacy questions *(practices)* • I feel comfortable communicating with patients who have substance use disorder, housing insecurity, and/or other structural vulnerabilities. • I am aware of and use the best practices in harm reduction when creating plans for patients with opiate use disorder, housing insecurity, or other structural vulnerabilities. • I am comfortable educating a patient at the time of discharge. • I feel confident in identifying structural factors which may increase a patient’s likelihood of leaving the hospital AMA.
Post-OSCE Written Reflections	- Written reflection essays *(attitudes*,* practices)* • Describe the dilemma you and your patient experienced. How would you describe this to a colleague and what language would you use? • Have you ever had a patient request to leave prematurely or “Against Medical Advice”? What happened? How was it different or similar to this experience? What were the structural factors that contributed to the patient’s decision or dilemma? • What concepts or strategies, if any did you employ in (a) communication with the patient and (b) developing a safe discharge plan? • What did you learn from this case? Do you foresee any ways you will change your approach to patients who request to leave prematurely after completing your coursework and OSCE? • What would you consider a “successful” outcome for a patient and physician in this situation?

AMA = Against Medical Advice, OSCE = objective structured clinical exam

**Fig. 1:**
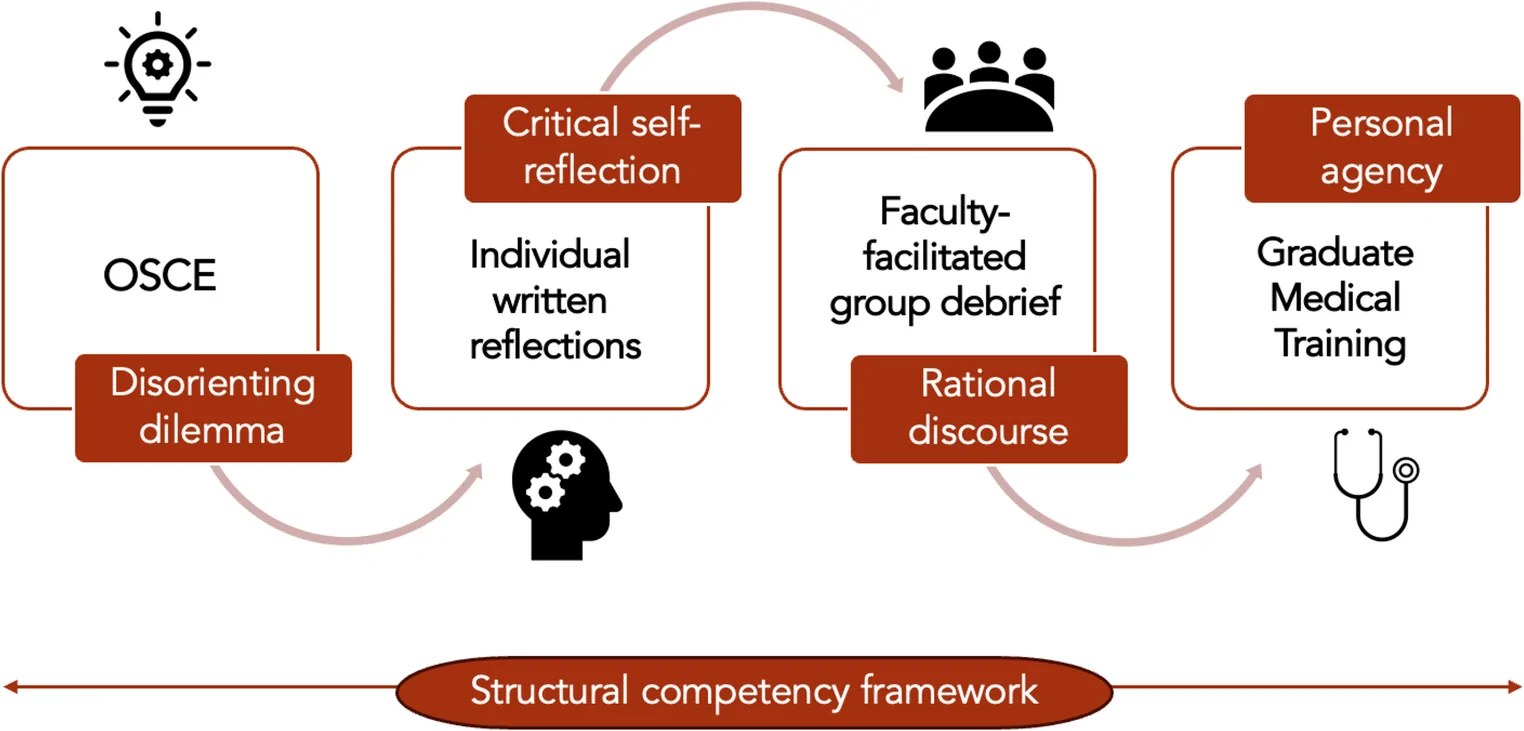
Conceptual diagram of the OSCE Curriculum and its alignment with the principles of transformative learning theory. Abbreviations: OSCE = objective structured clinical exam. Lines demonstrate the sequence of events in the curriculum, with labels below each box denoting how each event aligns with the principles of transformative learning. The line labeled “structural competency framework” highlights how structural competency teaching is incorporated throughout all elements of the curriculum

The components of the OSCE curriculum mirror the stages of TL, which posits that learners shift their worldview via several stages including (1) a disorienting dilemma, (2) critical self-reflection, (3) rational discourse, and (4) personal agency [12, 16]. During the OSCE, students are immersed in an emotionally and cognitively challenging situation, which serves as a disorienting dilemma. The simulation is followed by written reflection which pushes learners to engage in critical self-reflection and question prior assumptions. Students subsequently engage with peers and faculty to discuss their shared experiences and reflections, thus facilitating rational discourse. Finally, given the timing of the course and the proximity to entry into residency, students are positioned to practice personal agency and explore how they plan to adapt their behaviors to their new perspectives (Fig. 1).

### Setting, Participants, and Data Collection

Participants included fourth-year medical students enrolled in the required Health Policy course at Weill Cornell Medical College in New York City during academic year (AY) 2022–2023 and 2023–2024 (*n* = 190). Students were sent a pre-course survey via email accompanied by an in-person announcement at the start of the course. A post-survey was sent upon course completion and closed after two weeks. Student data was included if they completed both surveys. No incentives were provided to students. The Institutional Review Board at Weill Cornell Medical College approved this study (Study Number: 21–10024045).

Participating students were provided a link to a Qualtrics (Provo, UT) survey which prompted them to create a unique identifying code used to match surveys. The pre-survey included demographics, plans for residency, and clinical experiences. The pre- and post-surveys (Appendix 1) consisted of the 11-item Medical Condition Regard Scale [17] (MCRS) and four items assessing perceived self-efficacy in applying the learning objectives.

The MCRS is an 11-item validated score used to measure attitudes, including biases, emotions, and expectations, towards different conditions. The score “taps the degree to which medical students find patients with a given medical condition to be enjoyable, treatable, and worthy of medical resources” and has been reported to have good internal consistency (Crohnbach’s alpha 0.87) and construct validity [17]. The MCRS was originally validated for 12 medical diagnoses, somatoform conditions, and psychiatric diagnoses, and subsequently has been used to measure medical students’ attitudes towards patients with substance use disorders [17–20]. As there is currently no validated scale in the literature to measure attitudes towards patients who leave AMA, we adapted the MCRS to patients experiencing AMA discharge.

The four self-efficacy items, which used a 6-point Likert scale, measured students’ confidence in communicating with patients with structural vulnerabilities, using harm reduction when creating plans for patients with structural vulnerabilities, educating patients at discharge, and identifying structural factors that increase a patient’s likelihood of leaving the hospital prematurely, a key competency of the SC framework (Table 1).

To further explore self-reported changes in attitudes and practices, we qualitatively analyzed the written reflections from AY 2023–2024. Five reflection prompts (Table 1) were created to align with learning objectives for the curriculum and explored students’ experiences, how they applied the SC framework, and what they learned from the simulation, along with prior experiences with AMA discharge. Reflections were submitted to Canvas Learning Management System (Salt Lake City, UT). All reflection essays were de-identified, given a unique identifying number, and uploaded to MAXQDA (Berlin, Germany) qualitative analysis software. Qualitative analysis was done on reflections from consenting students after course grades were submitted.

### Data Analysis

Survey data for the MCRS and self-efficacy items were converted to numerical values and analyzed in SAS version 9.4 (SAS Institute, Cary, NC). MCRS scoring was completed according to prior description in the literature [17]. Paired t-tests were used to compare mean MCRS scores and mean self-efficacy items before and after the study intervention. Statistical significance was set at *P* < 0.05. Effect sizes were calculated using Cohen’s *d* to quantify the magnitude of the difference between groups [21]. To assess potential attrition bias, baseline demographic characteristics were compared between participants who completed both the pre- and post-surveys and those who completed only the pre-survey.

Researchers conducted an inductive thematic analysis following Braun and Clarke’s six-phase framework [22]. Of 62/108 (57.4%) consenting participants across three cohorts in AY 2023–2024, 30 reflections were randomly selected for qualitative analysis, with 10 reflections chosen from each cohort for a balanced representation. Authors familiarized themselves with the reflections and jointly inductively coded three reflections to develop a preliminary codebook. Authors SK and AR then independently coded batches of three reflections at a time, meeting regularly to reconcile all coding decisions. DO was available as a third coder to resolve any disagreements. The codebook was iteratively refined through these discussions. 18 reflections were coded in this fashion. Coding was comprehensive and not limited to the research question.

Researchers re-read the uncoded copies of the 18 previously coded reflections to re-immerse themselves in the data and identified eight codes (Appendix 2) that addressed the research question. Researchers then re-coded 18 essays with the eight codes in the revised codebook. To confirm that the eight codes captured all relevant ideas related to the research question, the team coded three additional reflections. No new codes were identified in the three reflections. Researchers then synthesized the codes into four themes. The team met to refine theme language and select representative quotations. Analysis of 21 reflections achieved sufficient information power [23] to answer the research question. All 21 coded reflections were randomly given a unique identifying number. To ensure the sampling strategy was representative of the broader dataset, authors SK and MAH read 16 additional uncoded reflections and concluded that the reflections echoed the four identified themes; no new themes were discovered. Quantitative data was analyzed separately and in parallel to qualitative data analysis. When data analysis was complete, authors converged the results by comparing, contrasting, and synthesizing findings.

### Reflexivity

Researchers AR, SK, and MAH are all hospitalist clinician educators familiar with the SC framework. AR was course director for the Health Policy course and SK is the director of the clinical skills center. DO is a research assistant with experience in qualitative methods. MAH, SK, and DO were not involved in student assessment. Researchers openly discussed how their own biases and experiences [24] shaped their understanding of student reflections.

## Results

### Quantitative Results

Of 190 eligible students over AYs 2022–2023 and 2023–2024, 77 (40.5%) completed the pre-survey and 50 (26.3%) completed both the pre- and post-surveys. One student did not complete the self-efficacy questions on the post-survey. Demographic characteristics are available in Table 2. There were no significant differences in demographics between participants who completed both surveys and those who completed only the pre-intervention survey (Appendix 3).

**Table 2:** Characteristics of medical student survey respondents (*n* = 50), Weill Cornell Medical College, New York, New York, 2023–2024

	*N* (%)
Age	
20–25	10 (20%)
26–30	36 (72%)
31–35	4 (8%)
Race/Ethnicity	
White	21 (42%)
Asian or Asian American	17 (34%)
Other^*^	12 (24%)
Gender (Female)	24 (48%)
Plans After Graduation	
Non-surgical residency	36 (72%)
Surgical residency	10 (20%)
Miscellaneous	4 (8%)
with AMA Experience	41 (82%)
with SUD Experience	50 (100%)

AMA = Against Medical Advice, SUD = Substance Use Disorder

*Other includes “Black, African, African American or Afro Caribbean” (n = 3), “Hispanic, Latino/Latina, or Spanish Origin” (*n* = 7), “Other” (*n* = 1), “Prefer not to answer” (*n* = 1)

The mean pre-course MCRS score (*n* = 50) was 4.18/6 (SD 0.79) and the mean post-course MCRS score was 4.57/6 (SD 0.75) showing a mean increase of 0.39 (SD 0.49, paired-samples t-test *P* < 0.001), indicating a statistically significant improvement in self-reported attitudes towards patients who leave AMA (Table 3). The standardized mean difference indicated large effect size (Cohen’s *d* = 0.81, 95% CI [0.49, 1.12]).

**Table 3:** Summary of quantitative data from medical student survey respondents (*n* = 50 for MCRS score and *n* = 49 for self-efficacy questions), Weill Cornell Medical College, New York, New York, 2023–2024

	Pre-Course Mean (SD)	Post-Course Mean (SD)	Mean difference (SD)	*P*-value	Cohen’s d (95% CI)
MCRS score (*n* = 50)	4.18 (0.79)	4.57 (0.75)	0.39 (0.49)	< 0.001	0.81 (0.49–1.12)
I feel comfortable communicating with patients who have substance use disorder, housing insecurity, and/or other structural vulnerabilities.^*^(*n* = 49)	4.57 (1.15)	4.94 (0.85)	0.37 (0.86)	0.003	0.43 (0.14–0.72)
I am aware of and use the best practices in harm reduction when creating plans for patients with opiate use disorder, housing insecurity, or other structural vulnerabilities.^*^ (*n* = 49)	3.51 (1.31)	4.82 (0.78)	1.31 (1.14)	< 0.001	1.15 (0.79–1.51)
I am comfortable educating a patient at the time of discharge.^*^(*n* = 49)	4.55 (1.08)	5.27 (0.64)	0.71 (1.02)	< 0.001	0.70 (0.39–1.01)
I feel confident in identifying structural factors which may increase a patient’s likelihood of leaving the hospital AMA.^*^ (*n* = 49)	4.10 (1.31)	5.33 (0.63)	1.22 (1.21)	< 0.001	1.01 (0.67–1.35)

MCRS = Medical Conditions Regard Scale

*Six-point Likert scale was employed: 1 = strongly disagree, 2 = disagree, 3 = not sure but probably disagree, 4 = not sure but probably agree, 5 = agree, and 6 = strongly agree

Students (*n* = 49) demonstrated statistically significant self-reported improvements across all four self-efficacy domains. They reported greater perceived comfort communicating with structurally vulnerable patients (*P* = 0.003; Cohen’s *d* = 0.43, 95% CI [0.14–0.72]), increased awareness of and use of the best practices in harm reduction when creating plans for patients with structural vulnerabilities (*P* < 0.001; Cohen’s *d* = 1.15, 95% CI [0.79–1.51]), improved comfort educating a patient at the time of discharge (*P* < 0.001; Cohen’s *d* = 0.70, 95% CI [0.39–1.01]), and improved confidence in identifying structural factors which may increase a patient’s likelihood of leaving the hospital AMA (*P* < 0.001; Cohen’s *d* = 1.01, 95% CI [0.67–1.35]) (Table 3).

### Qualitative Results

Qualitative analysis of 21 reflections identified four themes highlighting how students: (1) employed patient-centered communication strategies, (2) utilized best practices in discharge planning, (3) engaged in critical self-reflection and reframing, and (4) appreciated how structural barriers shape health.

#### Theme 1: Students Employed Patient-centered Communication Strategies with a Focus On Building Trust

Students reflected on using patient-centered communication strategies with the simulated patient, particularly when responding to the patient’s request to leave AMA. Strategies included using open-ended questions and attempts to build trust.

*During the first encounter*,* I asked open-ended questions to understand his rationale for leaving and how he was weighing the risks and benefits of that decision. To that aim*,* I asked what the ideal outcome would be in a perfect world*,* and then I worked towards reaching that goal or close to it. (437)*

Students used empathy and non-judgmental language to shift blame away from the patient and partner with them on common goals.

*I took time out of the conversation to ensure that she did not feel like anyone was blaming her for her situation. I told my patient that our team’s focus is on your health*,* but we know that it’s not that simple. That is why we want to make sure we are supporting you and the other things going on in your life to ensure you have a swift recovery. (113)*

Finally, students used a patient-centered approach to explore the patient’s perspective and identify competing priorities driving the patient to leave. Students recognized the power that giving the patient a voice has in developing a therapeutic alliance.

*Patients who feel heard by their providers are more likely to work with the team to facilitate the best plan*,* even if premature discharge is inescapable. In the future*,* I will always try to hear the patient’s side fully and try to understand what factors are at play requiring them to leave. (199)*

#### Theme 2: Students Utilized Best Practices in Discharge Planning Centered On Collaboration and Harm Reduction

After students developed a therapeutic rapport, they applied best-practice clinical skills. Students used a collaborative approach when developing the discharge plan and counseling the patient at discharge. Many found shared decision-making helpful in developing a collaborative patient-centered discharge plan.

*I learned how to approach a patient who wants to leave the hospital prior to completing our intended treatment plan and utilize shared decision making to come up with a new plan that reduces harm and maximize medical care while allowing the patient to attend other obligations. (156)*

Many students took a harm reduction approach to develop a safer discharge plan. They adapted their care plans and developed the best possible medical plan given the patient’s circumstances.

*Ultimately*,* a strategy of harm reduction was necessary as this appointment was simply too important for the patient’s overall health and well-being – the need for housing supersedes the need to continue IV antibiotics. Therefore*,* I made sure to convey all the signs to look out for if her condition is worsening and provided detailed instructions for medications and follow-up. (22)*

To further reduce harm, students performed best practices in discharge counseling including discussing red-flag symptoms, return-to care precautions, and use of the teach-back method.

#### Theme 3: Students Engaged in Critical Self-reflection and Reframing

The simulated encounter led students to deeply self-reflect. Several students described increased awareness of their own limitations, knowledge gaps, or emotional responses, and some envisioned more patient-centered approaches.

*From this case*,* I really learned to start to let go of my compulsion for perfectionism and “doing the most for the patient.” Changing my approach to this*,* I was able to take more of a harm reduction and longer-term perspective. I think this will change my future interaction with patients by helping me preserve a therapeutic alliance and provide patients with as many tools as possible instead of taking a paternalistic approach. (88)*

The increased self-awareness led some students to critically recognize the stigma that surrounds AMA discharge. Some went further and recognized their own harmful attitudes and assumptions towards patients who leave AMA.

*I came into the case with the expectation that the patient did not agree to any of the medical team’s recommendations. When I spoke to the patient during the first encounter*,* my assumption was challenged because he wanted to stay to continue his treatment*,* but he had competing responsibilities. (437)*

With recognition of their own stigma, some resolved to work towards unlearning bias in the future. Others reframed and saw the opportunity to meet patients where they are rather than seeing AMA discharges as a failures.

*I see myself approaching AMA situations from a less biased perspective in the future*,* one where I will meet the patient wherever they are at. (33)*

Finally, students challenged the traditional metrics of success in medicine and learned to redefine a successful outcome as one where they reduce harm and engage the patient in future care.

*I would consider a successful outcome one in which (1) the physician effectively communicated the risks and benefits of leaving prematurely*,* (2) the patient has a discharge plan that makes them as safe as possible*,* and (3) the patient feels comfortable and able to continue engaging with the healthcare system. (179)*

#### Theme 4: Students Appreciated How Structural Barriers Shape Health

When reflecting on the dilemma, many students shifted the focus away from blaming individual patients, a common approach in clinical settings when patients leave AMA. Instead, students identified structural barriers as key factors influencing patients’ decisions to leave.

*When describing this to my colleague*,* I would want to use language that focuses on the structural circumstances of my patient. I would want to emphasize that it’s not that the patient wants to leave; rather*,* it’s that the patient is forced by her circumstances to leave. (48)*

Some went further, recognizing that structural vulnerabilities create competing priorities which made remaining hospitalized difficult when there were urgent needs outside the hospital.

*Though physicians and providers may have a hard time seeing how any other priority could supersede completing a hospital stay/treatment course*,* patients have very real and competing priorities*,* often stemming from structural vulnerabilities. (199)*

Other reflections showed an even deeper understanding of how structural forces shape long-term health outcomes, with some students considering how their role as physicians in promoting health may take many forms.

*This was the key dilemma - the patient’s medical needs were at odds with their urgent social circumstances. This dichotomy is a stark reminder of how health is inextricably linked to social conditions. As a clinician*,* my role expanded beyond the confines of medical management to consider these broader determinants of health. (3)*

## Discussion

Participation in the OSCE curriculum was associated with improved self-reported medical student attitudes toward patients who leave AMA, increased perceived self-efficacy in caring for structurally vulnerable patients, and improved confidence in applying elements of the SC framework to a clinical encounter, such as identifying structural factors impacting health. Qualitative analysis of written reflections triangulated and enriched the quantitative findings by providing context for how self-reported attitudes and intended practices were impacted. Students described deep self-reflection which allowed them to recognize and challenge prior assumptions, and in some cases their own stigma toward patients who left AMA. These behaviors align with structural humility, which is the fifth competency of the SC framework [7]. Many went on to reframe a typically conflict-ridden encounter to be more patient-centered.

Consistent with quantitative results showing improvement in perceived self-efficacy with patient communication, qualitative reflections illustrated how students built therapeutic rapport using patient-centered communication skills including open-ended questions, non-judgmental language, and shared decision-making. Similarly, students reported significantly improved confidence in their skills in caring for structurally vulnerable patients, including applying harm reduction techniques and educating patients at the time of discharge.

Finally, qualitative findings supported the quantitative improvements in students’ perceived confidence in identifying structural factors contributing to AMA discharge. This aligns with the first competency of the SC framework, recognizing structural barriers which shape clinical interactions [7]. Some recognized how structural forces can impact long-term health outcomes and imagined how their roles as physicians may take many forms to promote the well-being of their patients, which is consistent with the fourth competency of the SC framework, observing and imagining structural interventions [7].

Our findings align with our prior work [11] which found that students performed well on the OSCE; most students explored why the patient requested to leave prematurely, counseled the patient on the risks of leaving, employed harm-reduction strategies, and were willing to partner with the patient on a plan. This reinforces our findings of improved self-reported efficacy in using patient-centered communication skills, harm-reduction skills, and best practices in discharge counseling. Additionally, students valued applying elements of the SC framework to clinical encounters.

Our findings also align with previous SC curricular evaluations in the literature. A 2017 curricular evaluation by Neff and colleagues [9] found that participating residents reported positive influence on their relationships with their patients in qualitative evaluations, as the curriculum helped them to shift blame away from their patients and build partnerships. A 2020 evaluation of an interdisciplinary SC curriculum [10] found that participants with clinical experience said the curriculum helped them blame their patients less for poor health outcomes or personal decisions influenced by structural factors.

To our knowledge, this is among the first clinically integrated SC curricula for advanced medical students which uses simulation and reflection to apply concepts from the SC framework to clinical care. Through both quantitative and qualitative findings, we showed that the curriculum improved students’ self-reported attitudes towards structurally vulnerable patients and how it influenced their intended practices, a critical gap in the previous literature [9, 25]. While there has been an increasing number of SC curricula developed and described in the literature, most are classroom, online, or service-learning based and do not place an emphasis on clinical skills training and simulation, unlike our curriculum. In a 2023 scoping review of SC curricula in health sciences education, none of the 34 curricula included in the study incorporated simulation [25]. Curricular evaluations have included evaluation of knowledge, skills, attitudes, and perceptions of the training [25, 26] as our evaluation did, though no other evaluations used a pre-post course validated attitudinal scale such as the MCRS.

The novel use of simulation and active reflection aligns our curriculum with the principles of TL and provides a possible mechanism of change for the observed outcomes. By integrating simulation, written self-reflection, and active discussion with faculty and peers, our curriculum provided the conditions that could facilitate the shift in perspective described by TL. A scoping review of TL in health professions education [27] suggests that clinical training offers opportunities for TL as trainees are immersed in unfamiliar settings, which aligns with the use of simulation in our curriculum. A review of TL in graduate medical education [16] (GME) found that TL is usually used for professionalism training and professional identity formation, with gaps identified in using TL in diversity, equity, and inclusion curricula. Though our curricula was developed for medical students, it is an example of how TL can be adapted to health equity curricula. By explicitly teaching our students the clinical skills relevant for taking care of structurally vulnerable populations and giving them the opportunity to apply these skills to a low-stakes simulated encounter, our curriculum equipped learners with both a framework to understand inequity and the tools to care for vulnerable patients.

Our work suggests that when teaching SC, there is value in teaching clinical skills relevant to caring for structurally vulnerable patients and allowing for immersive clinical experiences, including simulation and reflection to facilitate TL. We appreciate the timing of the curriculum; students were advanced clinical learners who could integrate new concepts into their clinical experiences and move beyond the hypothetical because they had a clinical foundation yet were still impressionable. Our findings potentially counteract prior observed trends that attitudes and intent to work with the underserved decline throughout medical school, which makes our findings compelling and highlights the need to examine if they can be replicated [28–30]. We recognize that simulation is resource-intensive and may be prohibitive in many settings. However, we see value in adapting lessons learned from this curriculum to learners at all stages of training at the bedside [31]. Additionally, there is promise in using artificial intelligence standardized patients to provide low-cost scalable access to simulation [32].

Our study, which presents self-reported data, is a single-site study without a control group, limiting its generalizability and causality argument. The survey response rate was low, attrition was measurable, and follow-up was short-term, which may limit the generalizability of the findings. However, quantitative findings were triangulated with qualitative data, and comparison of demographic characteristics between students who completed only the pre-survey and those who completed both the pre- and post-surveys did not identify substantial differences. We acknowledge that selection bias may have influenced which students contributed to quantitative data. We were unable to collect demographics for the qualitative data, which may also limit generalizability. While the MCRS was designed to assess regard for medical conditions, we applied it to patients who experienced the phenomenon of AMA discharge, which is novel, and may impact construct validity. Finally, we recognize that attitudes began generally high and the self-reported change in attitudes was modest, potentially reflecting ceiling effect, though reassuringly Cohen’s *d* group means differed by more than 0.8 standard deviations, indicating a substantial difference between groups. It has not been established what a clinically significant change in attitudes is, but small attitudinal shifts may be meaningful in advanced medical students.

Future work should explore the durability of self-reported attitudinal and perceived-practice change by following learners into their graduate medical training and independent practice. While the SC framework aims to encourage clinicians to imagine structural interventions on a societal scale, it is equally important to foster high regard towards underserved populations to promote engagement in this important work.

## Conclusions

Our findings suggest that integrating education on structural factors with clinical skills training, reinforced through simulation and guided reflection in line with the principles of TL, can enhance trainees’ perceived self-efficacy in caring for structurally vulnerable patients and improve their self-reported attitudes toward these patients.

## Data Availability

The datasets during and/or analyzed during the current study available from the corresponding author on reasonable request.

## Appendix 1: Surveys

**Pre-Course Survey**.

Please create an 8-character code which will be used as a participant ID to link your surveys. Your unique code will consist of the last two digits of your cell phone number, the first two letters of the city of your birth, the first two letters of your middle name*, and the last two digits of your zip code (as of 12/2022). Please create this code and list below.

Ex. for Lisa Susan Brown, cell: 365-780-9242, born in Columbus, current zip 11215. Lisa’s participant ID could be: 42CoSu15.

*If you do not have a middle name, you may use the first two letters of your mother’s name.

**Demographics**:

Age: 20–25, 25–30, 30–35, 35–40, 40+.

How do you describe yourself (please check all that apply):

- American Indian, Alaska Native, or Native American.
- Asian or Asian American.
- Black, African, African American or Afro Caribbean.
- Hispanic, Latino/Latina, or Spanish Origin.
- Native Hawaiian or Other Pacific Islander.
- White.
- Other (please describe).
- Prefer not to answer.

How do you describe yourself?

- Female.
- Male.
- Gender non-conforming.
- Different identity (please describe).
- Prefer not to answer.

Residency Plans: open-ended.

Have you had clinical experience taking care of a patient who left Against Medical Advice (AMA)? Yes/No.

Have you had clinical experience taking care of a patient with substance use disorder? Yes/No.

**Questions 1–11 refer to patients who leave the hospital Against Medical Advice (AMA).**

1. I prefer not to work with patients like this.

1 = strongly disagree.

2 = disagree.

3 = not sure but probably disagree.

4 = not sure but probably agree.

5 = agree.

6 = strongly agree.

2. Patients like this irritate me.

1 = strongly disagree.

2 = disagree.

3 = not sure but probably disagree.

4 = not sure but probably agree.

5 = agree.

6 = strongly agree.

3. I enjoy giving extra time to patients like this.

1 = strongly disagree.

2 = disagree.

3 = not sure but probably disagree.

4 = not sure but probably agree.

5 = agree.

6 = strongly agree.

4. Patients like this are particularly difficult for me to work with.

1 = strongly disagree.

2 = disagree.

3 = not sure but probably disagree.

4 = not sure but probably agree.

5 = agree.

6 = strongly agree.

5. Working with patients like this is satisfying.

1 = strongly disagree.

2 = disagree.

3 = not sure but probably disagree.

4 = not sure but probably agree.

5 = agree.

6 = strongly agree.

6. I feel especially compassionate toward patients like this.

1 = strongly disagree.

2 = disagree.

3 = not sure but probably disagree.

4 = not sure but probably agree.

5 = agree.

6 = strongly agree.

7. I wouldn’t mind getting up on call nights to care for a patient like this.

1 = strongly disagree.

2 = disagree.

3 = not sure but probably disagree.

4 = not sure but probably agree.

5 = agree.

6 = strongly agree.

8. I can usually find something that helps patients like this feel better.

1 = strongly disagree.

2 = disagree.

3 = not sure but probably disagree.

4 = not sure but probably agree.

5 = agree.

6 = strongly agree.

9. There is little I can do to help patients like this.

1 = strongly disagree.

2 = disagree.

3 = not sure but probably disagree.

4 = not sure but probably agree.

5 = agree.

6 = strongly agree.

10. Insurance plans should cover patients like this to the same degree that they cover patients with other conditions.

1 = strongly disagree.

2 = disagree.

3 = not sure but probably disagree.

4 = not sure but probably agree.

5 = agree.

6 = strongly agree.

11. Treating patients like this is a waste of medical dollars.

1 = strongly disagree.

2 = disagree.

3 = not sure but probably disagree.

4 = not sure but probably agree.

5 = agree.

6 = strongly agree.

12. I feel comfortable communicating with patients who have substance use disorder, housing insecurity, and/or other structural vulnerabilities.

1 = strongly disagree.

2 = disagree.

3 = not sure but probably disagree.

4 = not sure but probably agree.

5 = agree.

6 = strongly agree.

13. I am aware of and use the best practices in harm reduction when creating plans for patients with opiate use disorder, housing insecurity, or other structural vulnerabilities.

1 = strongly disagree.

2 = disagree.

3 = not sure but probably disagree.

4 = not sure but probably agree.

5 = agree.

6 = strongly agree.

14. I am comfortable educating a patient at the time of discharge.

1 = strongly disagree.

2 = disagree.

3 = not sure but probably disagree.

4 = not sure but probably agree.

5 = agree.

6 = strongly agree.

15. I feel confident in identifying structural factors which may increase a patient’s likelihood of leaving the hospital AMA.

1 = strongly disagree.

2 = disagree.

3 = not sure but probably disagree.

4 = not sure but probably agree.

5 = agree.

6 = strongly agree.

**Post-Course Survey**.

Please create an 8-character code which will be used as a participant ID to link your surveys. Your unique code will consist of the last two digits of your cell phone number, the first two letters of the city of your birth, the first two letters of your middle name*, and the last two digits of your zip code (as of 12/2022). Please create this code and list below.

Ex. for Lisa Susan Brown, cell: 365-780-9242, born in Columbus, current zip 11215. Lisa’s participant ID could be: 42CoSu15.

*If you do not have a middle name, you may use the first two letters of your mother’s name.

**Questions 1–11 refer to patients who leave the hospital Against Medical Advice (AMA).**

1. I prefer not to work with patients like this.

1 = strongly disagree.

2 = disagree.

3 = not sure but probably disagree.

4 = not sure but probably agree.

5 = agree.

6 = strongly agree.

2. Patients like this irritate me.

1 = strongly disagree.

2 = disagree.

3 = not sure but probably disagree.

4 = not sure but probably agree.

5 = agree.

6 = strongly agree.

3. I enjoy giving extra time to patients like this.

1 = strongly disagree.

2 = disagree.

3 = not sure but probably disagree.

4 = not sure but probably agree.

5 = agree.

6 = strongly agree.

4. Patients like this are particularly difficult for me to work with.

1 = strongly disagree.

2 = disagree.

3 = not sure but probably disagree.

4 = not sure but probably agree.

5 = agree.

6 = strongly agree.

5. Working with patients like this is satisfying.

1 = strongly disagree.

2 = disagree.

3 = not sure but probably disagree.

4 = not sure but probably agree.

5 = agree.

6 = strongly agree.

6. I feel especially compassionate toward patients like this.

1 = strongly disagree.

2 = disagree.

3 = not sure but probably disagree.

4 = not sure but probably agree.

5 = agree.

6 = strongly agree.

7. I wouldn’t mind getting up on call nights to care for a patient like this.

1 = strongly disagree.

2 = disagree.

3 = not sure but probably disagree.

4 = not sure but probably agree.

5 = agree.

6 = strongly agree.

8. I can usually find something that helps patients like this feel better.

1 = strongly disagree.

2 = disagree.

3 = not sure but probably disagree.

4 = not sure but probably agree.

5 = agree.

6 = strongly agree.

9. There is little I can do to help patients like this.

1 = strongly disagree.

2 = disagree.

3 = not sure but probably disagree.

4 = not sure but probably agree.

5 = agree.

6 = strongly agree.

10. Insurance plans should cover patients like this to the same degree that they cover patients with other conditions.

1 = strongly disagree.

2 = disagree.

3 = not sure but probably disagree.

4 = not sure but probably agree.

5 = agree.

6 = strongly agree.

11. Treating patients like this is a waste of medical dollars.

1 = strongly disagree.

2 = disagree.

3 = not sure but probably disagree.

4 = not sure but probably agree.

5 = agree.

6 = strongly agree.

12. I feel comfortable communicating with patients who have substance use disorder, housing insecurity, and/or other structural vulnerabilities.

1 = strongly disagree.

2 = disagree.

3 = not sure but probably disagree.

4 = not sure but probably agree.

5 = agree.

6 = strongly agree.

13. I am aware of and use the best practices in harm reduction when creating plans for patients with opiate use disorder, housing insecurity, or other structural vulnerabilities.

1 = strongly disagree.

2 = disagree.

3 = not sure but probably disagree.

4 = not sure but probably agree.

5 = agree.

6 = strongly agree.

14. I am comfortable educating a patient at the time of discharge.

1 = strongly disagree.

2 = disagree.

3 = not sure but probably disagree.

4 = not sure but probably agree.

5 = agree.

6 = strongly agree.

15. I feel confident in identifying structural factors which may increase a patient’s likelihood of leaving the hospital AMA.

1 = strongly disagree.

2 = disagree.

3 = not sure but probably disagree.

4 = not sure but probably agree.

5 = agree.

6 = strongly agree.

## Appendix 2: Codes

1. explore patient priorities/drivers.
2. non-judgmental, patient-centered language.
3. apply best practices/evidence-based practices for discharge.
4. apply harm reduction approach.
5. shared decision making.
6. critical self-reflection, bias awareness.
7. reframing AMA discharges, redefining success.
8. recognizing structural barriers to care.

## Appendix 3

**Table 4:** Characteristics of Medical Student Survey Respondents Who Completed Pre- and Post-Surveys (*N* = 50, study cohort) and Respondents Who Completed Pre-Survey Only (*N* = 27)

	Completed pre- and post-surveys, *N* = 50 (study cohort)	Completed pre-survey only, *N* = 27	
	*N* (%)	*N* (%)	*P*-value
Age			0.12
20–25	10 (20%)	1 (4%)	
26–30	36 (72%)	22 (81%)	
31–35	4 (8%)	4 (15%)	
Race/Ethnicity			0.5
White	21 (42%)	12 (44%)	
Asian or Asian American	17 (34%)	6 (22%)	
Other^*^	12 (24%)	9 (33%)	
Gender (Female)	24 (48%)	19 (70%)	0.06
Plans After Graduation			0.6
Non-surgical residency	36 (72%)	17 (63%)	
Surgical residency	10 (20%)	6 (22%)	
Miscellaneous	4 (8%)	4 (15%)	
with AMA Experience	41 (82%)	22 (81%)	0.96
with SUD Experience	50 (100%)	26 (96%)	0.17
Pre-course MCRS score, mean (SD)	4.2 (0.8)	4.1 (0.7)	0.63

*AMA* = Against Medical Advice, *SUD* = Substance Use Disorder

*Other includes “Black, African, African American or Afro Caribbean”, “Hispanic, Latino/Latina, or Spanish Origin”, “Other”, “Prefer not to answer”
